# Global, regional, and national burden of encephalitis from 1990 to 2021: result from the global burden of disease study 2021

**DOI:** 10.3389/fneur.2026.1652475

**Published:** 2026-02-12

**Authors:** Xinting Li, Bin Lu, Xi Deng, Haoran Liu, Xiaoli Luo

**Affiliations:** 1 Chengdu Women’s and Children’s Central Hospital, School of Medicine, University of Electronic Science and Technology of China, Chengdu, China; 2 Department of Pediatric Critical Medicine, Chengdu Women’s and Children’s Central Hospital, School of Medicine, University of Electronic Science and Technology of China, Chengdu, China; 3 Department of Pediatrics, Affiliated Hospital of Southwest Medical University, Luzhou, China

**Keywords:** ASDR, ASIR, ASMR, ASPR, disease burden, encephalitis, GBD 2021

## Abstract

**Background:**

Encephalitis is a life-threatening neurological disease with a major impact on global public health. This study uses the Global Burden of Disease 2021 (GBD 2021) data to assess the disease burden of encephalitis and provide evidence for targeted public health interventions.

**Methods:**

This study utilized the Global Burden of Disease (GBD) 2021 data analysis platform to examine trends in the prevalence, incidence, mortality, and disability-adjusted life years (DALYs) of encephalitis from 1990 to 2021, categorizing data by sex, age, and socio-demographic index (SDI). Dynamic patterns were analyzed at global, regional, and national levels. The Joinpoint Regression Program aided comparative analysis by examining annual percentage change (APC) and identifying significant turning points. Spearman’s rank correlation analysis quantified associations between the encephalitis burden and SDI.

**Results:**

From 1990 to 2021, global trends in encephalitis showed declines in prevalence, incidence, mortality, and DALYs. During this period, males had a higher age-standardized incidence rate (ASIR) and age-standardized mortality rate (ASMR) than females. Notably, children under five experienced the highest ASIR and age-standardized disability-adjusted life-year rate (ASDR), whereas individuals aged 95 and older had the highest ASMR. Additionally, case and age-standardized rate (ASR) varied significantly by geography, especially in lower SDI regions. At the national level, Pakistan, India, Bhutan, and Nepal faced a much higher disease burden. Finally, there was also a strong negative correlation between encephalitis ASR indicators and SDI.

**Conclusion:**

Overall, despite a declining global burden of encephalitis, significant disparities in disease burden persist across different countries and regions. This observation highlights a significant imbalance, underscoring the need for targeted public health strategies to effectively mitigate and address these disparities.

## Introduction

Encephalitis is a clinical neurological syndrome characterized by brain dysfunction associated with inflammation of the brain parenchyma (1). This condition is commonly triggered by infectious pathogens or induced through autoimmune processes, the latter potentially being post-infectious, paraneoplastic, or idiopathic (2). The breadth of infectious and autoimmune causes—numbering in the hundreds—shows the wide range of encephalitis presentations. Infectious etiologies typically present as acute febrile illnesses, accompanied by changes in personality, behavior, cognition, or consciousness. New-onset seizures or focal neurological deficits can also occur (3). Autoimmune encephalitis, in contrast, leads to varied symptoms such as seizures, behavioral changes, and cognitive decline. Immunosuppressive therapies are available, but many AE patients still face lasting cognitive impairment and refractory epilepsy after the acute phase, which lasts more than 3 months after diagnosis or treatment starts (4). Recently, climate change has sped up changes in vector transmission routes. Meanwhile, the growing use of immunosuppressive therapies and biologics—along with the increasing identification of autoantibodies related to autoimmune encephalitis—has made encephalitis a significant public health concern (5).

Encephalitis affects people of all ages, placing a heavy burden on patients, families, and society. Data show that both children and adults often face lasting effects after encephalitis. A review of outcomes in children found that almost half of survivors have ongoing neurodevelopmental issues, including cognitive delays, behavior changes, intellectual disability, and motor problems (6). In 26% to 62% of adult cases, serious aftereffects can significantly reduce quality of life, including seizures, memory loss, inappropriate behavior, poor social skills, fatigue, sleep disturbances, personality changes, cognitive issues, pain, sensory problems, and difficulties with daily activities (7). Encephalitis is a major public health challenge because of its frequency, death rate, and lasting complications.

The Global Burden of Disease (GBD) database provides a comprehensive set of quantitative tools designed to systematically assess the overall impact of various diseases, injuries, and their associated risk factors on the health status of global populations. This database not only supports cross-country and cross-temporal data comparisons but also possesses the capability to track the evolution of health indicators, thereby providing critical support for informed decision-making by public health policymakers. This paper systematically reviews and analyzes the development trends of the age-standardized prevalence rate (ASPR), age-standardized incidence rate (ASIR), age-standardized mortality rate (ASMR), and age-standardized disability-adjusted life-year rate (ASDR) for encephalitis globally, regionally, and by country from 1990 to 2021. Furthermore, the data were refined through multi-dimensional categorization based on age, gender, and socio-demographic index (SDI), enabling a comprehensive quantitative assessment of the disease burden of encephalitis. This systematic analysis not only reveals complex patterns in disease distribution but also provides a robust scientific foundation and guiding principles for developing precise and effective public health intervention strategies.

## Methods

### Data source

The GBD database is the world’s main and complete source of disease burden data. It utilizes data from various sources, including population counts, household surveys, official records, disease records, air quality monitors, and satellite images. You can get this data from the Institute for Health Metrics and Evaluation at the University of Washington (8). The GBD team utilizes three main standard tools: the Cause of Death Ensemble Model (CODEm), Spatiotemporal Gaussian Process Regression (ST-GPR), and Disease Model-Bayesian Meta-Regression (DisMod-MR) (9). CODEm is a highly systematic tool whose core functionality lies in employing multiple modeling strategies to estimate data for specific causes of death, covering mortality rates or cause-specific proportions, while flexibly selecting the most suitable covariates for validating out-of-sample prediction performance. ST-GPR is a suite of regression methods grounded in spatial and temporal associations, designed to estimate single indicators globally (10). DisMod-MR employs meta-regression analysis strategies to integrate multiple datasets, generating estimation parameters applicable to diverse population groups and geographic regions. This Bayesian geospatial software integrates a range of disease indicators, epidemiological frameworks, and spatial information to produce highly accurate and reliable assessment outcomes (9).

The GBD 2021 study conducted a comprehensive assessment of 371 types of diseases and injuries, as well as 88 risk factors, across 204 countries and regions worldwide (11). The Global Health Data Exchange query tool^1^ provides detailed information on the assessment of encephalitis. To generate the raw dataset, a systematic method was used. First, we accessed the data retrieval interface.^2^ Different assessment options were available in the “GBD Estimate” dropdown, with “Causes of death or injury” selected by default. In the “Measure” menu, we chose incidence, prevalence, mortality, and disability-adjusted life years. The “Metric” menu offered counts, percentages, and rates. The “Cause” menu listed diseases; we chose encephalitis for analysis. The “Location” menu provided detailed global and regional options: global, 5 SID regions, 21 super-regions, 204 countries, and more. Age and gender filters were available. The “Age and Gender” menu included an age-standardized option to ensure comparability across different age groups. The “Year” dropdown enabled selection from 1990 to 2021. The GBD2021 data adhere to open access principles for knowledge sharing and global health collaboration. To retrieve data, enter your query terms and click Search. Users can download CSV files with the “Download CSV” button.

### Case definition

In GBD 2021, encephalitis is defined as acute inflammation of the brain, with symptoms including headache, fever, drowsiness, fatigue, and sometimes seizures, hallucinations, or stroke. Encephalitis is coded in GBD modeling using ICD-10 codes A83-A85.2, A85.8-A86.0, B94.1, F07.1, G04-G05.8, and in the disease database by ICD-9 codes 062–064.9, 310.89, 323–323.9, and V05.0-V05.1.

### Socio-demographic index

The socio-demographic index (SDI) is a comprehensive indicator that measures a country’s socio-demographic development holistically. Its construction is based on key dimensions, including per capita income, educational attainment, and total fertility rate (12). The SDI value ranges from 0 to 1, with higher values indicating a higher level of socio-economic development (11). Based on 2021 SDI values, countries and regions are categorized into five distinct groups: Low SDI, Low-Middle SDI, Middle SDI, High-Middle SDI, and High SDI. The SDI values cited in this study encompass data from 21 regions spanning the period from 1990 to 2021. All data originate from official GBD resources,^3^ which are publicly accessible and downloadable.

### Statistical analysis

To comprehensively assess and compare the disease burden of epidemic encephalitis at global, regional, and national levels from 1990 to 2021, and to ensure comparability of analytical results, this study employs age-standardization using the standardized population from the GBD framework. This provides a unified assessment benchmark. Based on this, the age-standardized rates (ASRs) per 100,000 individuals are determined using the following formula:

$$\text{ASRs}=\frac{\sum_{i=1}^{A}a_{i}w_{i}}{\sum_{i=1}^{A}w_{i}}\times 100,000$$

where ai denotes the age-specific ratio in the i-th age group, w represents the number of individuals (or weight) in the corresponding i-th age group within the selected reference population, and a indicates the number of age groups.

To analyze and predict ASR trends, this study employed generalized linear models (GLMs) to calculate the estimated annual percentage change (EAPC) of ASR over a 30-year period. The EAPC was obtained by fitting a linear regression model to the natural log of the ASR, using calendar year as the independent variable. The model’s slope was transformed to yield the EAPC. The corresponding 95% uncertainty Interval (UI) was calculated to quantify the temporal variation of ASR. The EAPC formula is as follows:

EAPC=100×(exp(β)−1)

β represents the annual change in ASR per 100,000 population. EAPC is a widely used summary measure for assessing ASR trends over a specific period (13). A lower bound of the EAPC value and a 95% UI both greater than 0 indicate an increasing ASR trend. Conversely, an increase in both the EAPC value and the 95% UI, with a value less than 0, indicates a decreasing trend in ASR. When the 95% UI value equals 0, it indicates a constant ASR trend.

To assess trends in the burden of encephalitis from 1990 to 2021, we used Joinpoint regression (version 5.2.0, April 2024) to calculate Annual Percentage Changes (APCs) and their 95% Confidence Intervals (CIs). This approach quantitatively shows how trends change over time and ensures reliable statistical inferences. APCs significantly different from zero indicated increasing (worsening) or decreasing (improving) trends; no significant difference meant stable or flat trends (12). The nodal regression model enables a detailed analysis of trends in incidence rates by recognizing and combining multiple trend patterns. Compared to using a single trend line in methods like Poisson regression or time series models (14), this approach fits data sequences more precisely and captures the complexity of changes in incidence rates more thoroughly. Spearman correlation was used to examine the relationship between four metrics (ASPR, ASIR, ASMR, and ASDR) and SDI across 21 regions, aiming to identify factors that may influence the burden of encephalitis. All statistical analyses were performed using R software version 4.3.3. Results with *p* < 0.05 were considered statistically significant.

## Results

### Global burden of encephalitis

**Figure 1:**
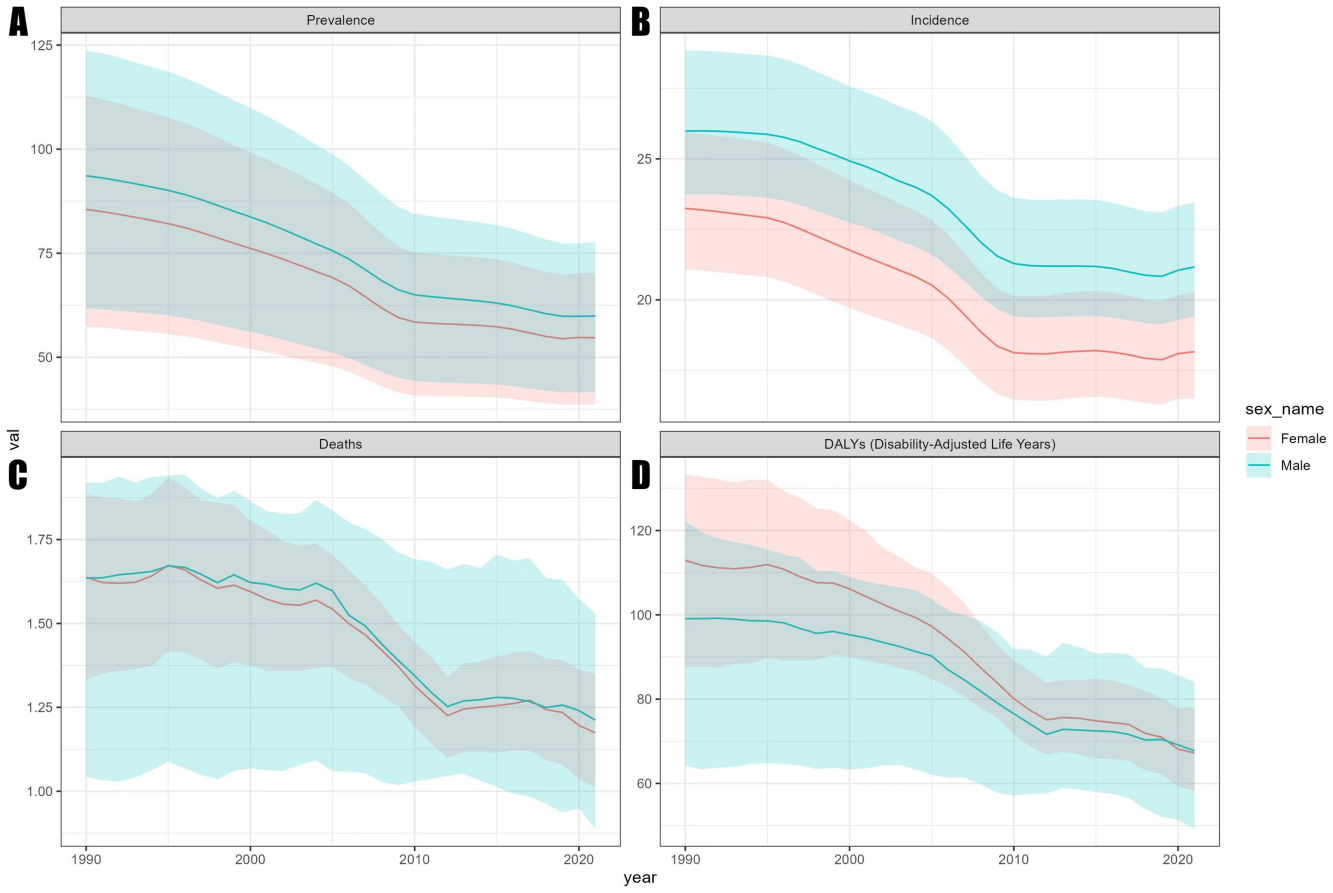
Trends in ASR of the global encephalitis burden by sex, 1990–2021. **(A)** ASPR. **(B)** ASIR. **(C)** ASMR. **(D)** ASDR.

**Figure 2:**
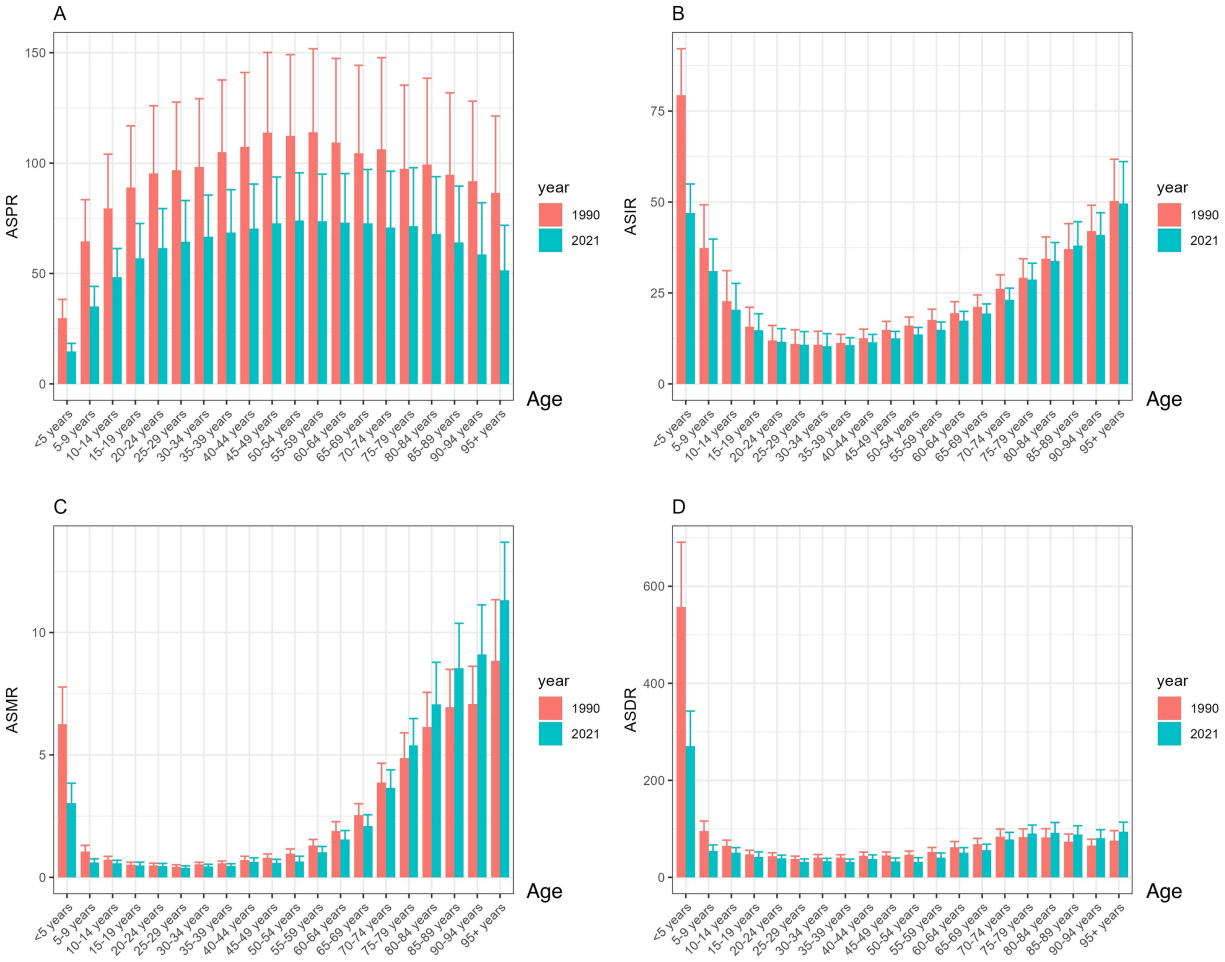
Trends in ASR of the global encephalitis burden by age, 1990–2021: **(A)** ASPR, **(B)** ASIR, **(C)** ASMR, and **(D)** ASDR.

Statistical data indicate that the total number of encephalitis cases in 2021 was 4643564.3 (3245326.9, 5999959.1). This includes 1492767.7 (1364664.3, 1654928.6) new cases, 91947.7 (78328.7, 106091.3) fatal cases, and 4952818.6 DALYs (4095518.4, 5697350.5) (Tables 1–4). Although the global total number of cases and deaths increased from 1990 to 2021, the global ASPR, ASIR, ASDR, and ASMR for encephalitis showed a general downward trend over the past three decades. Compared to 1990, the global ASPR decreased by 35.9% (32.0, 38.2) (Table 1). The ASIR decreased by 19.8% (18.3, 21.4) (Table 2). The ASMR decreased by 26.4% (8.4, 38.2) (Table 3). The ASR of death (ASDR) decreased by 36.1% (18.3, 47.9) (Table 4). Among these indicators, the decline in ASIR was more moderate compared to ASPR, ASMR, and ASDR. The EAPC for ASIR was −0.96 (−1.05, −0.86). The analysis of encephalitis burden from gender and age perspectives revealed that between 1990 and 2021, ASIR and ASPR for encephalitis were consistently higher among males than females, although ASMR did not show significant gender differences during this period. From 1990 to 2019, the ASDR for females exceeded that of males; however, after 2019, the male ASDR surpassed the female ASDR (Figure 1), indicating an inverted pattern of gender-related health effects. Age-stratified analysis showed that, while ASPR remained low among children under five (Figure 2A), ASIR and ASDR were elevated in this group (Figures 2B,D). Notably, ASDR peaked among children under five, declined with age, and rose again in older age groups. ASIR for children under five was markedly elevated in 1990 but showed a significant downward trend by 2021. The ASPR rose with increasing age, peaking in the 50–54 age group in 2021 before gradually declining. In contrast, in 1990, the ASPR peaked in the 55–58 age group and then declined sharply. Overall, ASPR data for 1990 were higher than in 2021 (Figure 2A), indicating a downward trend. Similarly, ASIR decreases with age, reaches a minimum in the 30–34 age group, then rises with age (Figure 2B). ASMR dynamics from 1990 to 2021 exhibit a declining trend during adulthood, with the lowest ASMR observed at 25–29 years, followed by an increase and a peak in the 95 + age group (Figure 2C). This study highlights a dynamic relationship between age and mortality risk: for those aged five and older, ASDR shows limited variation, but for children under five, ASDR is significantly higher than in other age groups (Figure 2D).

**Table 1:** Global prevalence numbers and ASPR of encephalitis, along with their temporal changes from 1990 to 2021.

Prevalence	1990		2021		Percentage change in the ASPR (95% UI)	EAPC (1990–2021)
	Number (95% UI)	ASPR (95% UI)	Number (95% UI)	ASPR (95% UI)		
Global	4625610.2 (3115878.4, 6,093,198)	89.3 (59.5, 117.9)	4643564.3 (3245326.9, 5999959.1)	57.3 (40.2, 73.9)	−35.9 (−38.2, −32)	−1.69 (−1.8, −1.59)
SDI						
High SDI	119,034 (84011.3, 153353.1)	12.8 (9.1, 16.4)	128559.5 (91140.6, 164316.7)	10 (7.3, 12.7)	−21.8 (−23.9, −19.5)	−0.53 (−0.65, −0.4)
High-middle SDI	586260.8 (404479.5, 763864.9)	54 (37.2, 70.3)	501891.7 (351671.7, 639319.5)	34.9 (24.9, 44.3)	−35.5 (−38.2, −32.2)	−1.37 (−1.43, −1.31)
Middle SDI	1737092.6 (1191232.2, 2267485.6)	106.5 (71.6, 139.9)	1563282.8 (1100691.9, 2004390.2)	60.8 (43, 78)	−42.9 (−45.3, −39.6)	−2.01 (−2.09, −1.92)
Low-middle SDI	1706667.3 (1,120,164, 2268086.3)	170.7 (109.5, 227.6)	1702835.7 (1,182,942, 2216196.3)	92.4 (63.2, 120.6)	−45.9 (−48.4, −41.7)	−2.37 (−2.54, −2.2)
Low SDI	475006.8 (308323.8, 636200.7)	119.6 (75, 160.9)	745294.9 (518204.1, 963,468)	82.6 (55.6, 107.5)	−30.9 (−34.5, −25.4)	−1.62 (−1.77, −1.46)
Region						
Andean Latin America	12315.6 (8470.6, 16127.7)	36.1 (24.3, 47.6)	17140.5 (12218.3, 21846.6)	26 (18.5, 33.1)	−28 (−32, −22.2)	−1.31 (−1.44, −1.17)
Australasia	444 (317, 577.2)	2.1 (1.5, 2.7)	689.7 (490.7, 875.9)	1.9 (1.4, 2.4)	−6.9 (−13.3, −0.5)	−0.29 (−0.36, −0.23)
Caribbean	16460.6 (11406.3, 21400.7)	48.5 (33.2, 63.4)	18479.2 (12782.2, 24012.1)	37.7 (26.2, 48.9)	−22.4 (−25.2, −19.6)	−0.96 (−1.11, −0.82)
Central Asia	27384.8 (19247.7, 35096.1)	43.3 (29.9, 56)	35025.5 (24928.4, 44472.4)	36.4 (25.7, 46.4)	−15.9 (−18.7, −12.8)	−1.16 (−1.41, −0.92)
Central Europe	23686 (16498.3, 30503.5)	17.9 (12.5, 22.9)	14993.3 (10450.9, 19307.8)	10.6 (7.6, 13.4)	−40.9 (−42.5, −38.9)	−1.86 (−1.97, −1.75)
Central Latin America	79479.4 (56,307, 102602.7)	51.1 (35.2, 66.7)	115196.7 (81,597, 148327.7)	44.6 (31.6, 57.3)	−12.8 (−15.4, −9.2)	−0.8 (−0.91, −0.69)
Central Sub-Saharan Africa	15661.1 (10042.2, 21243.1)	36.4 (22.6, 49.2)	42,833 (29109.6, 57188.8)	38.5 (25.4, 52)	5.6 (−2.7, 14.9)	−0.1 (−0.26, 0.05)
East Asia	1300603.9 (890434.1, 1707560.4)	106 (71.7, 139.5)	936659.7 (656848.7, 1206006.1)	59.4 (42.5, 75.6)	−44 (−47.1, −40)	−1.65 (−1.74, −1.56)
Eastern Europe	65468.5 (44676.2, 84680.6)	26.9 (18.6, 34.6)	56734.6 (38251.9, 73277.1)	22.9 (16, 29.4)	−14.6 (−16.4, −12.7)	−0.96 (−1.14, −0.79)
Eastern Sub-Saharan Africa	68517.1 (44289.1, 92250.5)	47.6 (29.3, 64.1)	141856.6 (97547.1, 184,510)	41.6 (27.6, 54.6)	−12.5 (−16.6, −4.8)	−0.48 (−0.52, −0.45)
High-income Asia Pacific	35358.7 (24,982, 45780.6)	19.1 (13.6, 24.6)	33534.4 (23655.8, 43443.6)	15.4 (11.1, 19.7)	−19.3 (−21.3, −17)	−0.21 (−0.45, 0.02)
High-income North America	8607.2 (5993.7, 11306.7)	2.8 (2, 3.7)	9379.1 (6518.7, 12027.8)	2 (1.4, 2.6)	−28.2 (−32.7, −23.7)	−1.52 (−1.72, −1.31)
North Africa and Middle East	71478.6 (49482.7, 93019.9)	24.5 (16.5, 32.3)	130123.5 (92713.2, 167122.5)	21.3 (15, 27.4)	−13.3 (−16.8, −7.9)	−0.72 (−0.8, −0.63)
Oceania	2443.3 (1641.5, 3239.9)	42.3 (27.4, 56.5)	4942.6 (3376.7, 6490.2)	38.6 (25.8, 50.9)	−8.8 (−14, −3.7)	−0.24 (−0.31, −0.17)
South Asia	2507843.8 (1657663.8, 3,306,977)	263.9 (170.7, 351.2)	2559989.1 (1788566.6, 3318759.1)	140.8 (97.2, 183)	−46.6 (−49, −42.4)	−2.52 (−2.73, −2.31)
Southeast Asia	228097.1 (155320.7, 299987.5)	53.4 (35.6, 70.6)	243827.9 (169217.2, 314333.4)	34 (23.6, 43.9)	−36.3 (−39, −32.6)	−1.67 (−1.83, −1.51)
Southern Latin America	7516.7 (5272.3, 9748.7)	15.4 (10.7, 20)	10781.6 (7573.2, 13936.7)	14.8 (10.5, 19)	−4.2 (−8.7, 0)	0.16 (−0.12, 0.44)
Southern Sub-Saharan Africa	13258.8 (8984.8, 17469.8)	29.4 (19.4, 39.1)	19919.3 (13088.7, 26430.9)	25.6 (16.6, 34.2)	−12.9 (−15.3, −10.7)	−0.5 (−0.53, −0.46)
Tropical Latin America	28339.6 (19,662, 36478.2)	20.3 (13.8, 26.3)	35213.3 (24644.5, 44932.9)	14.5 (10.2, 18.5)	−28.4 (−30.7, −25.4)	−2 (−2.36, −1.64)
Western Europe	43522.3 (30848.4, 55783.7)	10 (7.2, 12.8)	52839.7 (37,848, 66755.8)	9.7 (7.1, 12.1)	−3.8 (−6.6, −1)	−0.02 (−0.15, 0.12)
Western Sub-Saharan Africa	69123.2 (45486.7, 91456.7)	46.3 (29.6, 61.8)	163404.9 (112,239, 211983.2)	42.6 (28.5, 55.9)	−7.9 (−10.7, −3.4)	−0.45 (−0.5, −0.39)

**Table 2:** Global incidence numbers and ASIR of encephalitis, along with their temporal changes from 1990 to 2021.

Incidence	1990		2021		Percentage change in the ASIR (95% UI)	EAPC (1990–2021)
	Number (95% UI)	ASIR (95% UI)	Number (95% UI)	ASIR (95% UI)		
Global	1,356,348 (1228368.4, 1525339.8)	24.5 (22.3, 27.3)	1492767.7 (1364664.3, 1654928.6)	19.7 (18, 21.8)	−19.8 (−21.4, −18.3)	−0.96 (−1.05, −0.86)
SDI						
High SDI	39482.2 (34303.1, 45,565)	4.9 (4.2, 5.7)	48181.3 (43626.7, 53305.7)	4.5 (4, 5.2)	−7.1 (−10, −4)	−0.1 (−0.16, −0.04)
High-middle SDI	144748.2 (127228.9, 166850.3)	14.4 (12.7, 16.5)	143105.7 (129269.7, 159236.1)	12.9 (11.5, 14.8)	−10 (−13.2, −6.4)	−0.36 (−0.41, −0.32)
Middle SDI	440549.7 (395245.7, 500419.4)	25.1 (22.7, 28.1)	478616.9 (437088.4, 531532.9)	21.2 (19.4, 23.7)	−15.3 (−16.8, −13.7)	−0.75 (−0.83, −0.67)
Low-middle SDI	563993.3 (513189.8, 627277.8)	47.3 (43.7, 51.4)	579922.8 (533331.2, 636061.1)	32.5 (30.2, 35.3)	−31.2 (−32.9, −29.8)	−1.52 (−1.65, −1.39)
Low SDI	167054.3 (150634.9, 187688.7)	31.4 (28.8, 34.3)	242374.8 (219811.9, 271526.5)	23.2 (21.4, 25.2)	−26.2 (−27.8, −24.6)	−1.19 (−1.28, −1.1)
Region						
Andean Latin America	4801.1 (4324.6, 5370.2)	11.4 (10.4, 12.6)	6151.1 (5,576, 6791.7)	9.6 (8.7, 10.6)	−15.8 (−18.4, −12.7)	−0.72 (−0.79, −0.65)
Australasia	212.7 (186.2, 243.6)	1.1 (0.9, 1.2)	496.5 (447.7, 547.1)	1.4 (1.2, 1.5)	27.8 (20.5, 36.4)	0.91 (0.79, 1.03)
Caribbean	5,317 (4717.9, 6044.3)	14.4 (12.8, 16.2)	5327.6 (4734.5, 5989.2)	11.8 (10.5, 13.4)	−17.6 (−19.7, −15.6)	−0.56 (−0.68, −0.45)
Central Asia	11096.4 (9876.2, 12413.8)	14.7 (13.1, 16.2)	13,347 (12131.4, 14717.2)	13.8 (12.6, 15.2)	−5.7 (−7.8, −3.2)	−0.28 (−0.37, −0.19)
Central Europe	8174.1 (7215.4, 9135.2)	6.9 (6.1, 7.8)	6007.5 (5435.3, 6628.1)	5.1 (4.5, 5.8)	−26.1 (−28, −24.1)	−0.99 (−1.06, −0.93)
Central Latin America	30146.4 (25835.2, 35812.9)	15.7 (13.7, 18.2)	33,665 (29692.7, 38743.7)	14.6 (12.7, 16.9)	−7 (−9.2, −4.9)	−0.53 (−0.61, −0.44)
Central Sub-Saharan Africa	6029.3 (5192.9, 7046.2)	9.9 (8.6, 11.3)	12997.9 (11,119, 15263.7)	9.1 (7.9, 10.4)	−7.9 (−10.7, −5)	−0.26 (−0.3, −0.23)
East Asia	246528.1 (214646.1, 288357.3)	20.8 (18.1, 24.2)	218831.1 (194444.3, 247594.2)	18.9 (16.6, 22)	−9.3 (−13.7, −4.5)	−0.11 (−0.22, −0.01)
Eastern Europe	26142.6 (23559.8, 28931.2)	11.8 (10.6, 13.1)	22133.7 (20289.4, 24033.2)	10.7 (9.6, 11.8)	−9.4 (−11.3, −7.3)	−0.38 (−0.43, −0.33)
Eastern Sub-Saharan Africa	25250.3 (22108.2, 28934.5)	12.5 (11.2, 13.9)	47061.7 (41326.7, 54102.7)	11.5 (10.3, 12.8)	−8.3 (−9.6, −6.8)	−0.27 (−0.29, −0.25)
High-income Asia Pacific	10416.4 (8891.8, 12354.9)	6.9 (5.8, 8.3)	9843.8 (8735.6, 11047.3)	6.6 (5.6, 7.9)	−5.1 (−7.6, −2.4)	0.13 (−0.02, 0.29)
High-income North America	5121.3 (4,330, 5949.9)	1.8 (1.5, 2.1)	7369.9 (6563.1, 8146.4)	1.7 (1.5, 1.8)	−7.2 (−13.9, 1.4)	−0.4 (−0.48, −0.31)
North Africa and Middle East	31063.7 (26971.8, 36196.1)	8.6 (7.6, 9.7)	47040.3 (41413.3, 53830.2)	7.8 (6.9, 8.9)	−8.8 (−10.5, −7)	−0.38 (−0.41, −0.35)
Oceania	841.4 (720, 1005.6)	11.5 (10.1, 13.4)	1649.5 (1417.4, 1950.8)	11.1 (9.6, 12.9)	−3.6 (−7, −0.2)	−0.13 (−0.15, −0.11)
South Asia	804614.4 (731456.3, 890104.3)	73.3 (67.9, 79.5)	860218.6 (796883.1, 939528.5)	51.3 (47.8, 55.7)	−30 (−31.3, −28.8)	−1.53 (−1.7, −1.37)
Southeast Asia	72841.4 (63965.9, 85036.9)	15.1 (13.4, 17.1)	84275.2 (75841.7, 94192.7)	13 (11.7, 14.6)	−13.7 (−15.9, −11.5)	−0.65 (−0.73, −0.56)
Southern Latin America	2535.4 (2186.4, 2936.4)	5.1 (4.4, 5.9)	3748.4 (3327.9, 4,194)	5.8 (5.1, 6.6)	14 (9.7, 19.5)	0.7 (0.41, 0.98)
Southern Sub-Saharan Africa	5,741 (4,911, 6743.2)	10.5 (9.2, 12)	7879.2 (6855.2, 9045.1)	10 (8.8, 11.3)	−5.4 (−7, −3.8)	−0.21 (−0.32, −0.09)
Tropical Latin America	10855.7 (9613.7, 12282.4)	7 (6.3, 7.8)	12329.7 (11128.2, 13719.2)	5.6 (5.1, 6.3)	−20.1 (−23, −17.1)	−1.53 (−1.8, −1.25)
Western Europe	17171.3 (15132.5, 19293.2)	4.5 (3.9, 5.1)	23816.6 (21676.1, 26111.3)	4.9 (4.3, 5.5)	10 (7.1, 13.6)	0.44 (0.34, 0.53)
Western Sub-Saharan Africa	31447.8 (27655.8, 36113.2)	15 (13.4, 16.7)	68577.4 (60273.3, 78264.4)	13.9 (12.5, 15.5)	−7 (−8.7, −5.4)	−0.2 (−0.23, −0.16)

**Table 3:** Global death numbers and ASMR of encephalitis, along with their temporal changes from 1990 to 2021.

Deaths	1990		2021		Percentage change in the ASMR (95% UI)	EAPC (1990–2021)
	Number (95% UI)	ASMR	Number (95% UI)	ASMR		
Global	84188.8 (66553.7, 95321.8)	1.6 (1.3, 1.8)	91947.7 (78328.7, 106091.3)	1.2 (1, 1.4)	−26.4 (−38.2, −8.4)	−1.18 (−1.33, −1.03)
SDI						
High SDI	1977.9 (1882.2, 2073)	0.2 (0.2, 0.2)	3817.9 (3508.3, 4032.8)	0.2 (0.2, 0.2)	6.4 (−0.5, 12.1)	0.16 (−0.09, 0.42)
High-middle SDI	8771.4 (7373.6, 9955.8)	0.9 (0.8, 1)	6386.1 (5910.5, 7100.2)	0.5 (0.4, 0.5)	−47.9 (−54.9, −36.4)	−2.52 (−2.67, −2.37)
Middle SDI	26053.3 (20028.3, 29647.7)	1.7 (1.3, 1.9)	25669.3 (21706.4, 29764.5)	1.1 (0.9, 1.3)	−32.4 (−42.8, −17.2)	−1.42 (−1.57, −1.26)
Low-middle SDI	33297.9 (25770.6, 39277.8)	3.6 (2.8, 4.2)	39330.9 (31820.3, 47214.9)	2.7 (2.2, 3.2)	−26 (−40.6, −1.1)	−1.18 (−1.37, −0.98)
Low SDI	14048.7 (9911.2, 17659.1)	2.7 (2.2, 3.3)	16706.9 (13505.6, 19958.4)	1.9 (1.6, 2.3)	−30.6 (−43.8, −9.8)	−1.22 (−1.32, −1.12)
Region						
Andean Latin America	578.4 (465.6, 703)	1.3 (1.1, 1.6)	541.7 (414.1, 699)	0.9 (0.7, 1.1)	−34.9 (−51.2, −15.5)	−1.71 (−2.05, −1.36)
Australasia	14.7 (13.7, 15.7)	0.1 (0.1, 0.1)	79.8 (71.3, 87.5)	0.2 (0.2, 0.2)	155 (130.3, 180.1)	3.56 (3.02, 4.1)
Caribbean	351.6 (315, 398.3)	1 (0.9, 1.1)	204.1 (168.2, 247.9)	0.4 (0.4, 0.6)	−54.3 (−62.4, −44.5)	−2.15 (−2.81, −1.49)
Central Asia	1002.8 (866.8, 1173.9)	1.3 (1.1, 1.5)	942.9 (789.9, 1112.9)	1 (0.8, 1.2)	−23.6 (−39.6, −6.4)	−1.28 (−1.68, −0.88)
Central Europe	809.5 (764.6, 846.9)	0.7 (0.7, 0.8)	562.2 (515, 615.7)	0.4 (0.4, 0.4)	−45.9 (−50.8, −39.9)	−2.59 (−2.95, −2.22)
Central Latin America	1, 322 (1244.7, 1404.6)	0.7 (0.7, 0.8)	1606.8 (1, 423, 1834.3)	0.7 (0.6, 0.8)	−9.3 (−21, 4.3)	−0.29 (−0.64, 0.07)
Central Sub-Saharan Africa	317.1 (213.3, 486.3)	0.6 (0.4, 0.8)	570.4 (413, 750.7)	0.5 (0.4, 0.7)	−10.6 (−39.8, 23.6)	−0.35 (−0.41, −0.29)
East Asia	12382.1 (8541.7, 15175.5)	1.1 (0.8, 1.3)	3735.3 (3026.4, 4780.1)	0.3 (0.3, 0.4)	−69.4 (−77.9, −50.1)	−4.43 (−4.79, −4.06)
Eastern Europe	2342.1 (2260.1, 2422.6)	1.1 (1, 1.1)	2122.3 (1949.8, 2288.3)	0.9 (0.8, 0.9)	−18.9 (−25.5, −12.5)	−1.16 (−1.4, −0.92)
Eastern Sub-Saharan Africa	2434.4 (1654.5, 3003.1)	1 (0.7, 1.2)	3642.7 (2601.6, 4568.1)	0.9 (0.6, 1.1)	−14.6 (−35.4, 8.9)	−0.53 (−0.59, −0.47)
High-income Asia Pacific	402.9 (379.5, 467.8)	0.2 (0.2, 0.3)	687 (593.7, 751.8)	0.2 (0.2, 0.2)	−12.2 (−29.3, −5)	−1.24 (−1.7, −0.76)
High-income North America	359.4 (343.1, 369.2)	0.1 (0.1, 0.1)	985.3 (904.1, 1035.7)	0.2 (0.2, 0.2)	62.6 (55.2, 69.4)	1.94 (1.38, 2.5)
North Africa and Middle East	2491.7 (1999.1, 3047.8)	0.8 (0.6, 0.9)	2870.2 (2355.5, 3, 493)	0.5 (0.4, 0.6)	−29.1 (−41.6, −12.9)	−1.09 (−1.13, −1.05)
Oceania	23.5 (15.9, 34.3)	0.4 (0.3, 0.8)	59.7 (35.5, 96.1)	0.4 (0.3, 0.9)	14.5 (−22.6, 66.5)	0.48 (0.4, 0.56)
South Asia	46996.3 (37171.6, 54827.6)	5.6 (4.5, 6.5)	55087.5 (45273.1, 68076.9)	3.9 (3.2, 4.7)	−31.6 (−44, −7.9)	−1.46 (−1.67, −1.25)
Southeast Asia	6433.1 (4655.5, 8106.4)	1.4 (1.1, 1.7)	7883.2 (5611.2, 9524.9)	1.3 (0.9, 1.6)	−6.9 (−30.5, 17.6)	−0.18 (−0.26, −0.1)
Southern Latin America	192.1 (182.7, 202.4)	0.4 (0.4, 0.4)	299.3 (279.6, 320.4)	0.4 (0.4, 0.4)	1.3 (−7.1, 9.8)	0.1 (−0.43, 0.63)
Southern Sub-Saharan Africa	204.2 (144.7, 237)	0.5 (0.3, 0.5)	308 (225.4, 376.9)	0.4 (0.3, 0.5)	−6.3 (−25, 15.2)	−0.22 (−0.58, 0.14)
Tropical Latin America	465.8 (430.2, 502.1)	0.3 (0.3, 0.3)	593.8 (551.9, 633.4)	0.3 (0.2, 0.3)	−18.8 (−26.4, −10.8)	−0.51 (−0.7, −0.31)
Western Europe	777.6 (749.6, 800.7)	0.2 (0.2, 0.2)	2126.6 (1920.6, 2241.7)	0.3 (0.3, 0.3)	62.6 (55.7, 68.8)	1.72 (1.56, 1.88)
Western Sub-Saharan Africa	4287.5 (2887.6, 5965.6)	1.9 (1.4, 2.3)	7038.9 (4827.8, 8939.3)	1.5 (1, 1.8)	−20.6 (−42.1, 6.2)	−0.68 (−0.82, −0.53)

**Table 4:** Global DALYs and ASDR of encephalitis, along with their temporal changes from 1990 to 2021.

DALYs	1990		2021		Percentage change in the ASDR (95% UI)	EAPC (1990–2021)
	Number (95% UI)	ASDR (95% UI)	Number (95% UI)	ASDR (95% UI)		
Global	6065443.4 (4742546.8, 6962738.7)	105.5 (83.4, 120)	4952818.6 (4095518.4, 5697350.5)	67.4 (54.9, 78.4)	−36.1 (−47.9, −18.3)	−1.65 (−1.79, −1.51)
SDI						
High SDI	106371.2 (100045.9, 114503.5)	13.4 (12.6, 14.6)	125072.6 (117395.1, 133069.1)	11.1 (10.4, 11.9)	−17.4 (−22.9, −12)	−0.57 (−0.76, −0.38)
High-middle SDI	655062.3 (539020.1, 755817.3)	67.4 (55, 78.2)	321803.7 (292836.7, 364541.6)	29.3 (26.1, 34.6)	−56.5 (−63.4, −44.6)	−3.1 (−3.26, −2.94)
Middle SDI	1991737.2 (1523408.5, 2273682.7)	108.6 (84.1, 123.4)	1354832.2 (1135350.9, 1572906.6)	63 (51.5, 72.5)	−41.9 (−52.8, −27.8)	−1.94 (−2.09, −1.79)
Low-middle SDI	2224665.3 (1724381.3, 2660206.6)	173.8 (138.3, 202.1)	1976317.3 (1590550.8, 2394514.1)	110.5 (89.3, 134)	−36.4 (−49.6, −15)	−1.69 (−1.87, −1.5)
Low SDI	1084753.7 (719638.8, 1397251.2)	156.4 (115.7, 193.1)	1172608.9 (919834.3, 1402369.4)	94.8 (78.4, 111.6)	−39.4 (−52.3, −17.2)	−1.74 (−1.84, −1.65)
Region						
Andean Latin America	46168.4 (36664.8, 56624.7)	95.4 (77.2, 115.3)	34893.9 (26537.7, 45307.7)	54.8 (41.6, 71.3)	−42.6 (−59, −24.1)	−2.11 (−2.48, −1.75)
Australasia	634.5 (592.5, 681.5)	3.3 (3.1, 3.6)	2304.9 (2118.5, 2475.4)	7.2 (6.6, 7.9)	116.5 (93.7, 143.5)	3.07 (2.53, 3.62)
Caribbean	25786.7 (22, 713, 29968.6)	66.7 (59.2, 76.9)	13800.4 (11256.3, 17, 058)	31.8 (25.4, 40)	−52.3 (−60.9, −40.7)	−2.12 (−2.72, −1.51)
Central Asia	78291.2 (67491.5, 91366.9)	93.7 (81.3, 108.8)	65930.8 (54836.9, 78842.7)	67.1 (56, 80.1)	−28.3 (−43.3, −11.6)	−1.49 (−1.9, −1.07)
Central Europe	52757.1 (49870.3, 55603.9)	50.7 (47.7, 53.8)	22573.5 (20716.7, 24873.3)	20.9 (18.8, 23.2)	−58.8 (−63.1, −53.8)	−3.46 (−3.84, −3.08)
Central Latin America	108732.1 (101659.2, 117213.5)	53.5 (50.4, 57.4)	98473.2 (85642.4, 114031.6)	41.6 (36, 48.8)	−22.4 (−33.5, −9.3)	−0.84 (−1.17, −0.51)
Central Sub-Saharan Africa	24737.5 (16502.8, 40428.8)	33.7 (24.3, 48.5)	42534.2 (30937.4, 55490.8)	28.7 (21.9, 36.7)	−14.9 (−46.9, 17.9)	−0.5 (−0.58, −0.42)
East Asia	1135326.7 (800682.6, 1390430.7)	97.1 (68, 119.3)	304, 060 (252528.2, 377, 483)	29.6 (24.1, 38.9)	−69.5 (−77.4, −51.2)	−4.39 (−4.7, −4.08)
Eastern Europe	132705.9 (127235.7, 137969.1)	65 (62.2, 67.9)	86611.1 (79785.5, 93132.6)	43.9 (40.6, 46.8)	−32.5 (−37.8, −27.4)	−1.67 (−1.84, −1.51)
Eastern Sub-Saharan Africa	200822.4 (135606.2, 249450.3)	67.2 (48.1, 80.7)	284400.6 (204580.6, 359433.7)	54.6 (40.5, 67.4)	−18.7 (−39.8, 8.1)	−0.68 (−0.74, −0.62)
High-income Asia Pacific	20841.2 (19140.5, 24573.1)	13 (12, 15.6)	20524.7 (18735.1, 22366.4)	10.4 (9.5, 11.4)	−20 (−35.5, −13.5)	−1.37 (−1.78, −0.96)
High-income North America	15864.7 (15459.1, 16296.6)	5.8 (5.7, 5.9)	30197.5 (28690.4, 31470.1)	8 (7.6, 8.5)	38.9 (32, 46.3)	1.51 (1.01, 2.02)
North Africa and Middle East	184900.6 (146561.7, 232935.3)	44.9 (36.3, 54.8)	178296.5 (147, 275, 217824.1)	29.7 (24.6, 36.1)	−33.9 (−47.1, −18.6)	−1.35 (−1.41, −1.29)
Oceania	2020.5 (1065.4, 3038.7)	24.8 (18.1, 34.2)	4977.2 (2453.4, 8323.2)	29.7 (18.9, 46.2)	19.7 (−20.7, 84.1)	0.62 (0.49, 0.74)
South Asia	3103892.2 (2393984.5, 3689112.7)	266.2 (213.5, 308.8)	2621657.4 (2130838.6, 3238911.7)	158.1 (128, 196.7)	−40.6 (−52.3, −18.1)	−1.96 (−2.18, −1.74)
Southeast Asia	500046.7 (348918.7, 652875.5)	93.6 (67.2, 119.1)	495464.8 (331571.8, 607968.8)	80.4 (52, 99.5)	−14.1 (−38.8, 12.4)	−0.47 (−0.53, −0.4)
Southern Latin America	10900.4 (10322.5, 11524.3)	22 (20.8, 23.2)	13298.2 (12418.8, 14, 255)	20.6 (18.9, 22.3)	−6.3 (−14.3, 2)	−0.18 (−0.71, 0.35)
Southern Sub-Saharan Africa	14015.4 (10274.2, 16172.2)	25.3 (18.7, 29.1)	18895.2 (14111.7, 23238.1)	23.7 (17.8, 29)	−6.3 (−24.7, 14.6)	−0.23 (−0.54, 0.08)
Tropical Latin America	37453.5 (34399.5, 40825.8)	23.6 (21.8, 25.7)	30780.8 (27977.7, 33779.4)	14.7 (13.1, 16.3)	−38 (−45.9, −29.4)	−1.48 (−1.64, −1.32)
Western Europe	34030.3 (32543.4, 35620.7)	9.2 (8.8, 9.6)	59, 798 (56427.5, 62373.5)	11.7 (11.2, 12.3)	27.2 (22.7, 32.2)	0.9 (0.77, 1.04)
Western Sub-Saharan Africa	335515.4 (211699.2, 474092.9)	116.9 (82.4, 157.9)	523345.6 (358836.7, 672698.9)	86.1 (61.2, 107.8)	−26.3 (−47.1, −0.5)	−0.87 (−1.06, −0.68)

### Burden of SDI on encephalitis

**Figure 3:**
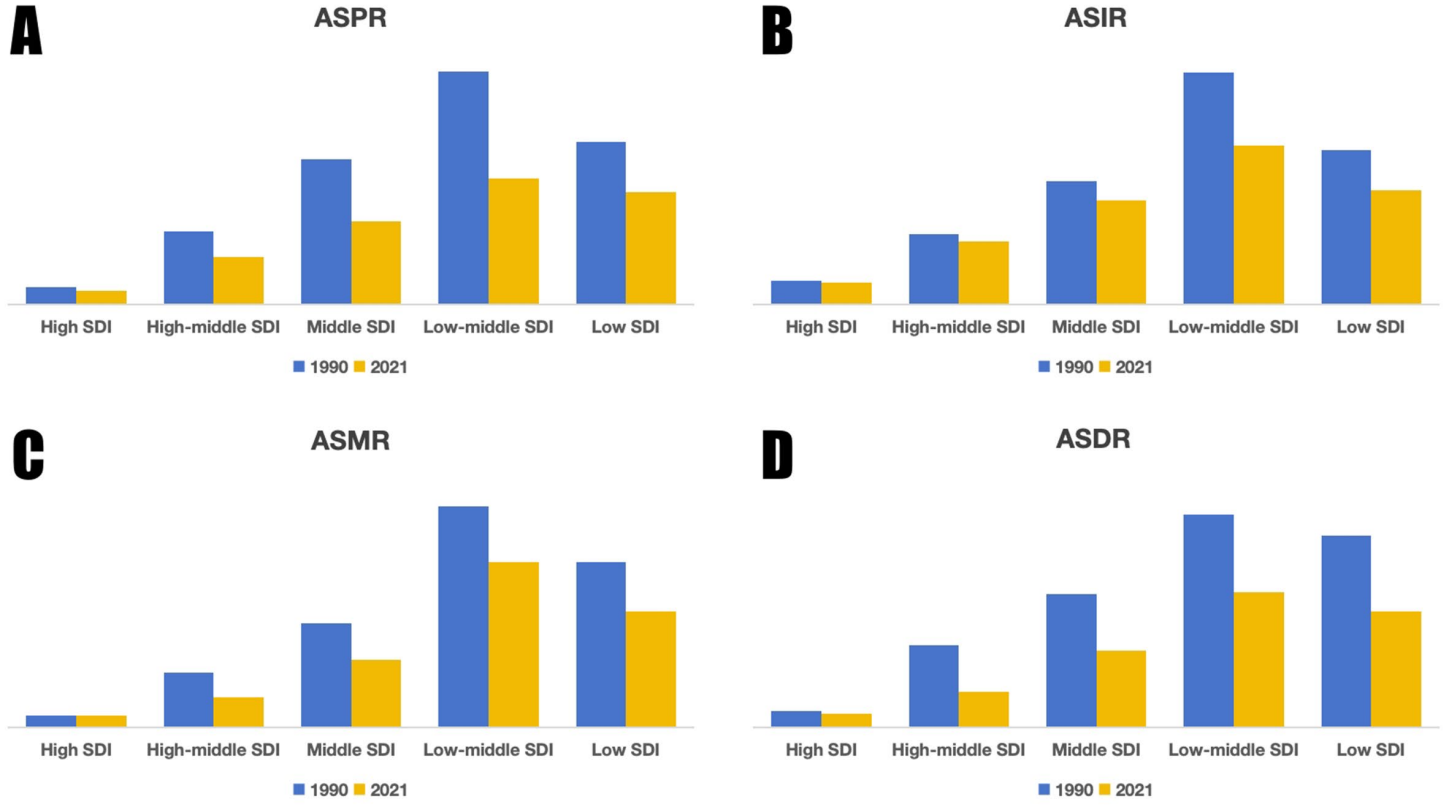
Trends in ASR of the global encephalitis burden by SDI, 1990 and 2021: **(A)** ASPR, **(B)** ASIR, **(C)** ASMR, **(D)** ASDR.

The SDI has a significant impact on the global distribution of encephalitis. In 2021, 4,625,610.2 (3,115,878.4, 4,609,319.8) cases occurred worldwide. Over 80% of these cases occurred in countries with medium and lower SDI (Table 1), indicating significant regional disparities. These gaps are clear in incidence, mortality, and DALYs. ASPR, ASIR, ASMR, and ASDR for encephalitis varied widely by SDI region. Specifically (Tables 1–4), low-middle SDI regions had the highest ASPR, ASIR, ASMR, and ASDR, while high SDI regions had the lowest values for these indicators (Figure 3). Compared to 1990, ASMR increased in high SDI regions by 6.4% (−0.5, 12.1), with an EAPC of 0.16 (−0.09, 0.42). Conversely, ASPR, ASIR, and ASDR declined across all five SDI regions (Figure 3). Low SDI regions experienced the largest decline in ASPR, decreasing by 45.9% (41.7, 48.4) with an EAPC value of −2.37 (−2.54, −2.2). Low-middle SDI regions saw the steepest ASIR reduction at 31.2% (29.8, 32.9), with an EAPC value of −1.52 (−1.65, −1.39). The ASMR in High-middle SDI regions decreased the most by 47.9% (36.4, 54.9), with an EAPC value of −2.52 (−2.67, −2.37). The greatest reduction in ASDR occurred in the High-middle SDI region at 56.5% (44.6, 63.4), with an EAPC value of −3.1 (−3.26, −2.94).

### Regional burden of encephalitis

At the regional level, South Asia recorded the highest burden of encephalitis in 2021, with the highest levels of ASPR, ASIR, ASMR, and ASDR, and has been identified as a region with a typical high burden. Moving to temporal trends, between 1990 and 2021, ASPR declined in all regions except Central Sub-Saharan Africa. Notably, South Asia experienced the largest decrease at 46.6% (42.4, 49), while Western Europe showed the smallest reduction at 3.8% (1, 6.6) (Table 1). Turning to changes in ASIR, rates increased in Australasia, Southern Latin America, and Western Europe, with the largest rise in Australasia at 27.8% (20.5, 36.4). In contrast, from 1990 to 2019, the remaining 18 GBD regions showed a significant downward trend in ASIR. During this period, South Asia experienced the largest decrease, at 30.0% (28.8, 31.3), while Oceania had the smallest decrease, at 3.6% (0.2, 7.0) (Table 2). Additionally, Australasia, High-income North America, and Western Europe were the three GBD regions with the lowest ASIR in 2021, ranging from 1.4 to 4.9 per 100,000 person-years. Regarding ASMR, these increased in Australasia, High-income North America, Oceania, Southern Latin America, and Western Europe, with Australasia showing the largest increase at 155% (130.3, 180.1). Meanwhile, ASMRs decreased in the remaining 16 regions. In 2021, South Asia, Southeast Asia, and Western Sub-Saharan Africa were the three GBD regions with the highest ASMR (exceeding 1.3). Importantly, all encephalitis ASMR in these regions has significantly declined over the past 30 years (Table 3). Last, except for increases in ASDR in Australasia, high-income North America, Oceania, and Western Europe, ASDR decreased in the remaining 17 regions. Specifically, East Asia experienced the largest decline at 69.5% (51.2, 77.4), while Southern Latin America and Southern Sub-Saharan Africa recorded the smallest decreases at 6.3% (3.2, 14.0) and 6.3% (14.6, 24.7), respectively (Table 4).

### National burden of encephalitis

**Figure 4:**
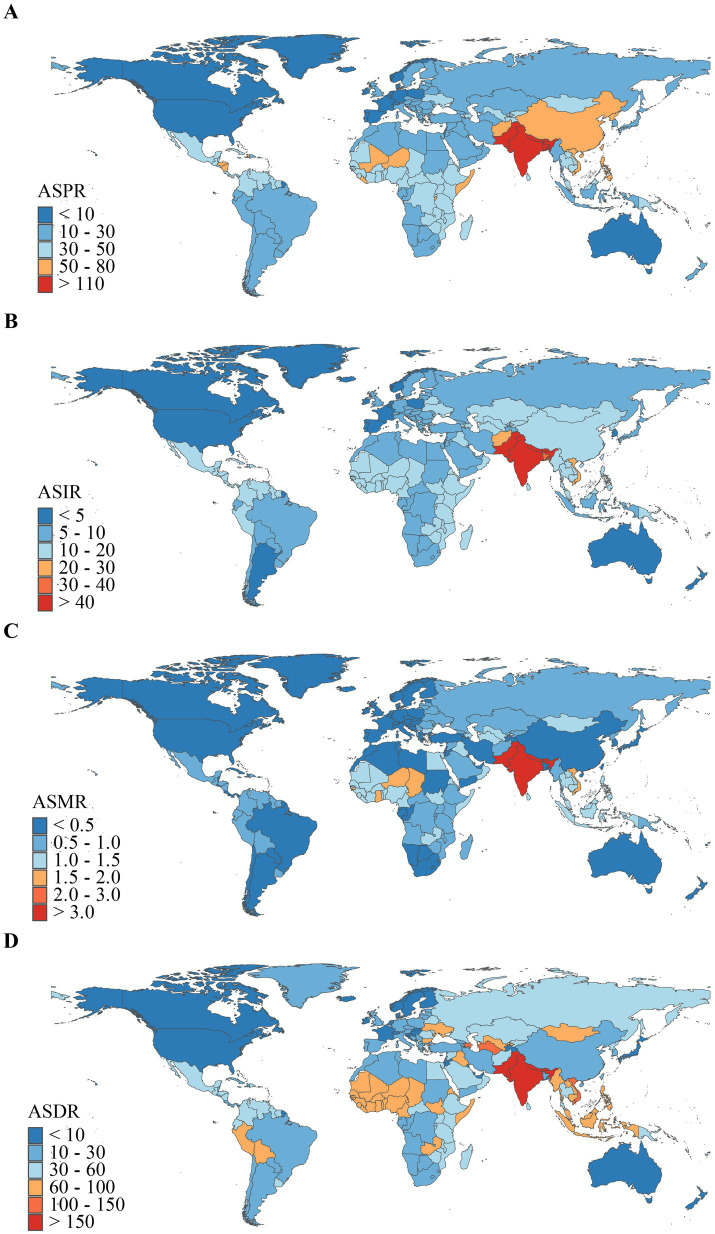
Geographical distribution of ASR of encephalitis in 2021. **(A)** ASPR. **(B)** ASIR. **(C)** ASMR. **(D)** ASDR.

In 2021, at the national level, the ASPR range for the burden of encephalitis across 204 countries was approximately 0.13 to 145.20 per 100,000 population (Figure 4A). Among all countries, Pakistan [145.20 (97.87–190.48)], India [143.72 (99.34–186.26)], and Nepal [138.20 (96.97–177.57)] exhibited the highest ASPRs. Notably, the top three countries are all located in South Asia, consistent with regional trends. Conversely, Canada, Australia, and Greenland had the lowest ASPR at [0.13 (0.09–0.17)], [0.30 (0.21–0.37)], and [0.49 (0.35–0.63)], respectively. In 2021, global ASIR for encephalitis ranged from 0.62 to 54.76 (Figure 4B), exhibiting corresponding geographic clustering patterns. Countries with the highest ASIR burden for encephalitis were Pakistan [54.76 (50.75–59.58)], India [53.45 (49.76–57.79)], and Bhutan [52.41 (48.47–56.83)]. Conversely, the countries with the lowest ASIR for encephalitis were Iceland [0.62 (0.54–0.71)], Australia [0.63 (0.55–0.72)], and Canada [0.72 (0.58–0.87)]. In 2021, the global ASMR for encephalitis ranged from 0.01 to 4.60 (Figure 4C). Countries with the highest ASMR included Pakistan [4.60 (2.25–6.48)], India [4.19 (3.49–5.26)], and Bhutan [4.08 (2.18–5.63)]. Conversely, countries with the lowest ASMR for encephalitis were the United States Virgin Islands [0.01 (0.00–0.01)], Malta [0.01 (0.01–0.01)], and Palau [0.01 (0.01–0.02)]. In 2021, the global ASDR for encephalitis ranged from 0.47 to 190.64 (Figure 4D). Countries with the highest ASDR included Pakistan (190.64, 100.93–268.21), India (169.82, 136.34–216.87), and Bhutan (164.78, 89.45–237.70). Conversely, countries with the lowest ASDR for encephalitis were Iceland [0.47 (0.42–0.54)], Malta [0.89 (0.75–1.05)], and Luxembourg [1.73 (1.47–2.02)]. It is evident that Pakistan, India, Bhutan, and Nepal exhibited relatively high disease burdens for encephalitis among the 204 GBD countries in 2021, with Pakistan ranking highest in ASPR, ASIR, ASMR, and ASDR.

### Correlation with sociodemographic indicators at the regional level

**Figure 5:**
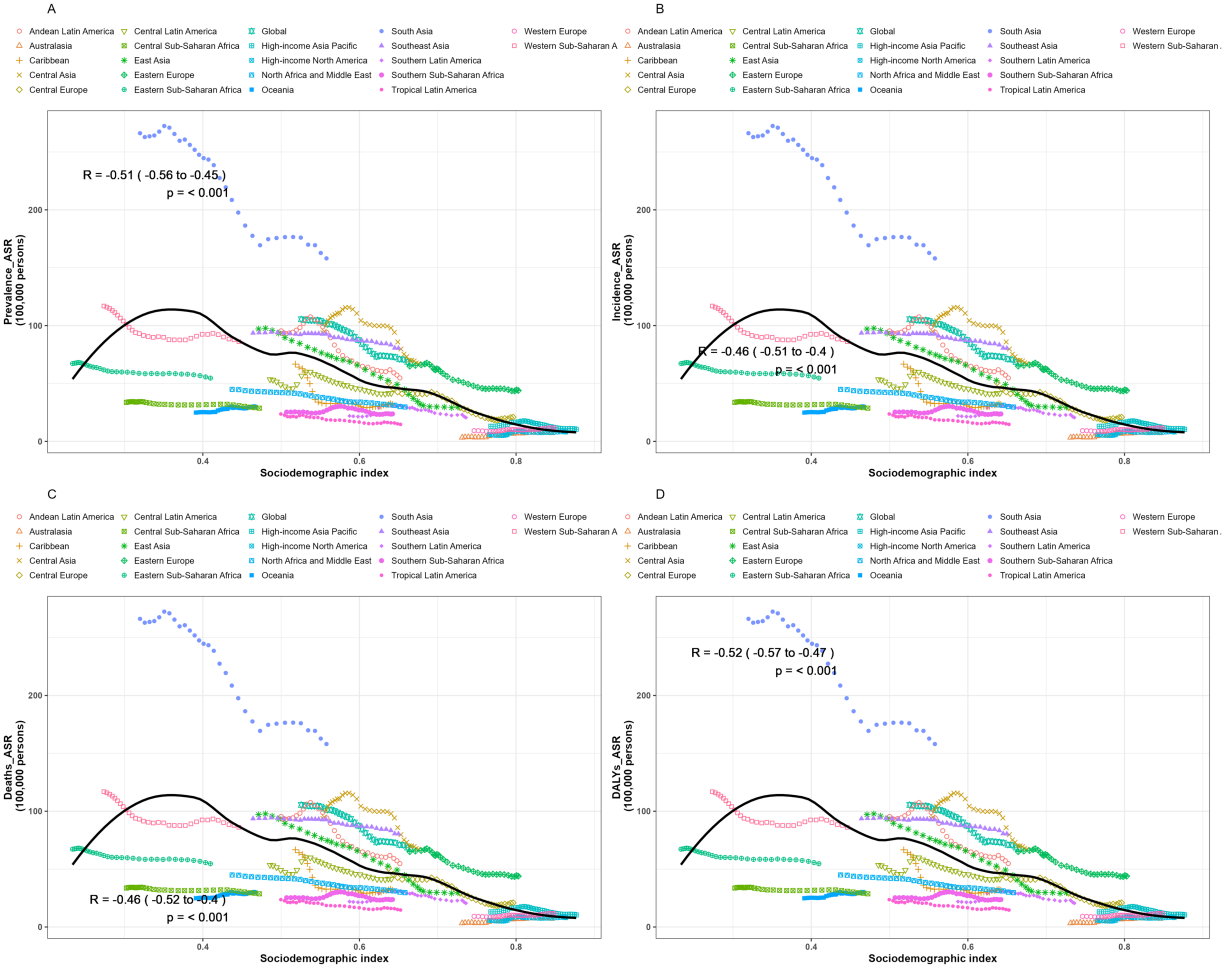
Trends and correlation analysis of ASR and SDI, 1990–2021. **(A)** ASPR. **(B)** ASIR. **(C)** ASMR. **(D)** ASDR.

We conducted a Spearman rank correlation analysis to examine the interrelationships between SDI values and ASPR, ASIR, ASMR, and ASDR across 21 regions from 1990 to 2021. Results (Figure 5) revealed a consistent negative correlation between encephalitis ASR and SDI across GBD regions, indicating lower encephalitis burden in areas with higher SDI. Regions with lower SDI exhibited observed encephalitis burdens that exceeded expected levels, while in regions with higher SDI, observed burdens were below expected. Analysis showed significant negative correlations between SDI and ASPR [*r* = −0.51 (−0.56, −0.45), *p* < 0.001], ASIR [*r* = −0.46 (−0.51, −0.4), *p* < 0.001], ASMR [*r* = −0.46 (−0.52, −0.4), *p* < 0.001], and ASDR [*r* = 0.52 (−0.57, −0.47), *p* < 0.001], indicating these rates decrease as SDI increases. Particularly in South Asia, the observed encephalitis burden significantly exceeded levels predicted based on SDI. However, throughout the study, the observed epidemic encephalitis burden was below expected levels in sub-Saharan Africa, Oceania, North Africa, the Middle East, southern Latin America, tropical Latin America, sub-Saharan Africa, Australia, and high-income North America. The reported burden of encephalitis in South Asia, Central Asia, Latin America in the Andean region, Southeast Asia, and Eastern Europe substantially exceeded levels predicted by their respective SDI. Overall, disease burden indicators such as ASPR, ASIR, ASMR, and ASDR typically exhibit a decreasing trend with rising SDI, reflecting a general reduction in disease burden within the context of economic development. However, the magnitude of this reduction varies significantly across different regions.

### Connection point analysis

**Figure 6:**
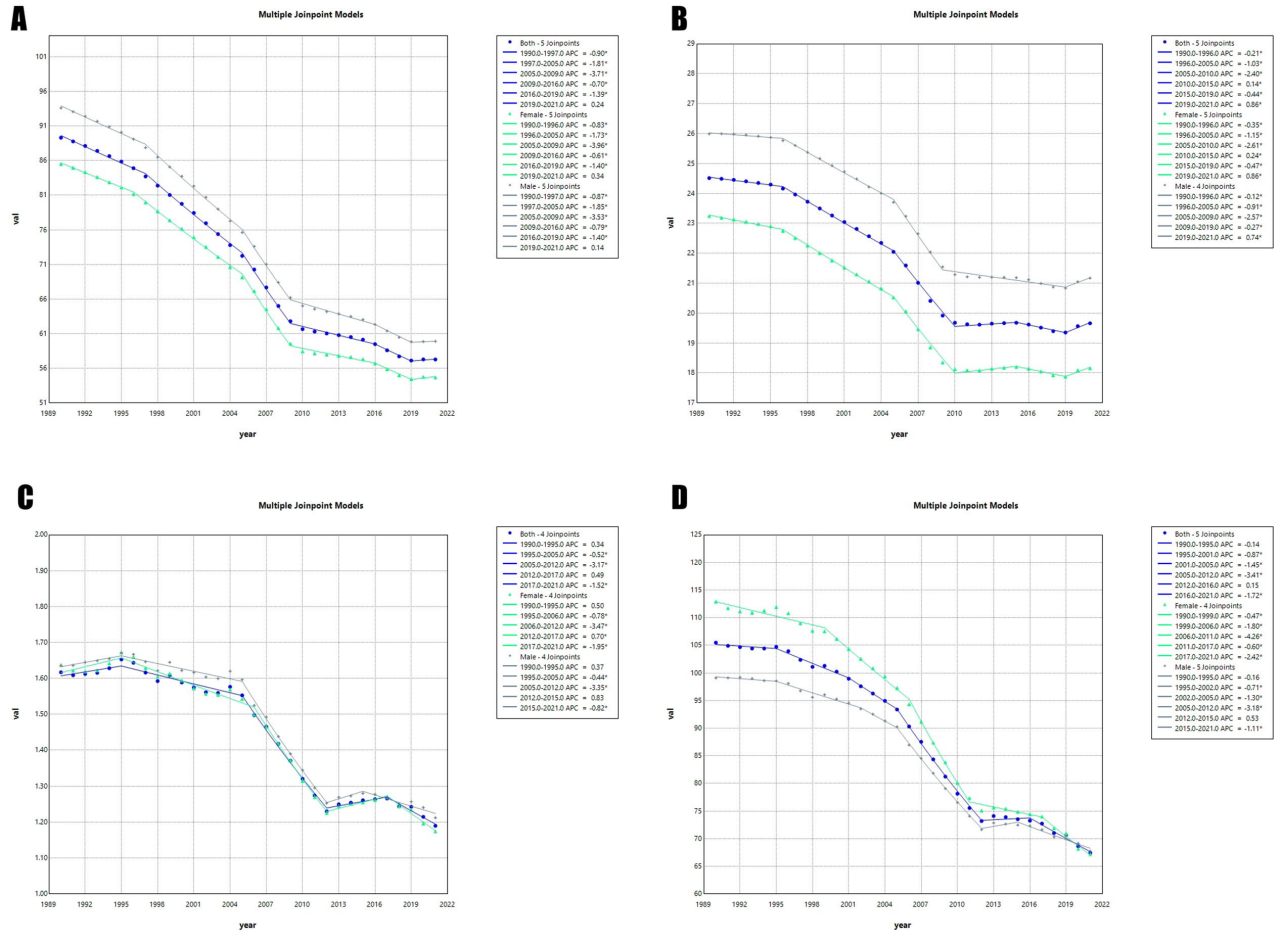
Jointpoint regression analyses of time trends in the global burden of encephalitis, 1990–2021: **(A)** ASPR, **(B)** ASIR, **(C)** ASMR, and **(D)** ASDR.

The jointpoint regression model can identify critical turning points in disease progression. By doing so, it segments the overall trend into distinct intervals. This enables the precise calculation of annual percentage changes within each segment. The result is a detailed quantitative analysis and understanding of the disease evolution process. In the chart (Figure 6), each segment between two jointpoints shows the trend of disease progression during that period. Turning points are typically associated with public health events. We conducted joint inflection point regression analyses on the ASPR, ASIR, ASMR, and ASDR of global encephalitis from 1990 to 2021. This allowed us to comprehensively evaluate its trends and variations. Analysis of the ASPR of global encephalitis from 1990 to 2021 (Figure 6A) shows an overall declining trend between 1990 and 2019. The annual percentage changes were: 1990–1997 = −0.90, 1997–2005 = −1.81, 2005–2009 = −3.71, 2009–2016 = −0.70, 2016–2019 = −1.39. The most pronounced decline occurred between 2005 and 2009 (APC = −3.71). Since 2019 (APC 2019–2021 = 0.24), this indicator has shown a slight resurgence. Analysis of ASIR for global encephalitis from 1990 to 2021 (Figure 6B) also reveals a declining trend from 1990 to 2010. The relevant APCs were: 1990–1996 = −0.21, 1996–2005 = −1.03, 2005–2010 = −2.40. A turning point occurred in 2010. From 2010 to 2015, the indicator increased slightly (APC 2010–2015 = 0.14). Between 2015 and 2019, a slight decline was observed (APC 2015–2019 = −0.44). Since 2019, the indicator has resumed a moderate upward trajectory (APC 2019–2021 = 0.86). Analysis of global ASMR for encephalitis between 1990 and 2021 (Figure 6C) reveals a modest upward trend from 1990 to 1995 (APC 1990–1995 = 0.34). However, from 1995, the indicator declined until 2012 (APC 1995–2005 = −0.52, APC 2005–2012 = −3.17). After 2012, this trend slightly reversed, showing a modest increase until 2017 (APC 2012–2017 = 0.49). From 2017 to 2021, the indicator again declined (APC 2017–2021 = −1.52). For the ASDR of global encephalitis from 1990 to 2021 (Figure 6D), a significant downward trend was observed between 1990 and 2010. The APCs were: 1990–1995 = −0.14, 1995–2001 = −10.87, 2001–2005 = −1.45, 2005–2012 = −3.41. From 2012 on, the indicator began a slight upward trend, rising until 2016 (APC 2012–2016 = 0.15). However, this rise was unsustainable. Between 2016 and 2021, the indicator declined again (APC 2016–2021 = −1.72), showing an overall pattern of complex and fluctuating development.

## Discussion

This study analyzes the progress achieved in encephalitis prevention and control at global, regional, and national levels over the past three decades. It systematically incorporates key factors such as age, gender, and SDI. This approach enables a more comprehensive understanding of the disease’s overall epidemiological patterns. The goal is to develop targeted interventions that address complex public health challenges in a scientifically effective manner. This will help lay a solid foundation for safeguarding human health and continuously strengthening global public health security.

To effectively assess and monitor the disease burden of encephalitis, a thorough analysis of its epidemiological characteristics is crucial. Notably, from 1990 to 2021, the ASIR of encephalitis worldwide showed a marked downward trend, a change closely linked to significant advances in precision medicine and enhanced resource allocation and strategy optimization in public health. Furthermore, the implementation of appropriate and timely treatment protocols is crucial for enhancing patient outcomes. Among infectious encephalitis cases, viral encephalitis accounts for approximately 70% of all confirmed diagnoses, marking it as the predominant type. In immunocompetent individuals, one of the primary causative agents of sporadic viral encephalitis is herpes simplex virus type 1 (HSV-1), which typically accounts for 10 to 20% of all viral encephalitis cases (15). The introduction of acyclovir revolutionized the clinical management of HSV encephalitis. Specifically, this medication competitively inhibits HSV replication by blocking deoxythymidine triphosphate (dTMP), a substrate for DNA polymerase, thereby disrupting DNA chain synthesis (16). Before the widespread implementation of specific antiviral therapies, herpes simplex encephalitis carried a mortality rate as high as 70%, leaving many survivors at risk of severe neurological sequelae. In contrast, since the introduction of acyclovir into clinical practice, the mortality rate for this disease has significantly decreased to 28%, markedly improving patient prognosis and quality of life. Notably, prompt initiation of antiviral treatment strategies can substantially reduce annual mortality rates to 14% within a short timeframe (17). The diagnosis of encephalitis is especially complex and challenging. This is particularly true for immunocompromised patients, whose bodies host a greater variety of infectious agents. It is also difficult with emerging infectious diseases, as clinicians may not recognize specific causative pathogens. New laboratory technologies offer promise for accelerating and optimizing diagnostic workflows. The adoption of neuroimaging techniques, such as cranial magnetic resonance imaging (MRI) and computed tomography (CT) scans, has greatly improved lesion localization. Meanwhile, innovative pathogen detection methods, including polymerase chain reaction (PCR) and metagenomic next-generation sequencing (mNGS), have broadened the spectrum of detectable pathogens and increased sensitivity. Together, these advances have improved the accuracy and reliability of encephalitis diagnosis (18). Medical innovations and technological advances have greatly improved the early diagnosis and timely treatment of encephalitis. As a result, ASIR has been steadily declining worldwide. In public health, coordinating key strategies and measures has decisively reduced both the incidence and ASR of encephalitis. Public health agencies focus on accelerating vaccine development and expanding vaccination campaigns. Evidence suggests that these strategies significantly reduce the risk of viral infection and protect vulnerable populations. For example, in Nepal, a five-year vaccination program aimed at preventing Japanese encephalitis resulted in a 78% reduction in infection cases, according to project data (19). Under the 2004 German public health policy framework, a comprehensive varicella vaccination initiative was implemented for one-year-old children. The implementation of this program significantly reduced the incidence of varicella-related neurological complications, with estimates indicating a reduction of up to 60% (20). This national experience demonstrates how targeted public health interventions can have a positive impact on neurological outcomes. Similarly, on a global scale, the reduction in the burden of encephalitis has primarily resulted from breakthroughs in medical technology innovation and their application in clinical practice, as well as the careful planning and effective execution of public health strategies.

Resource-constrained countries and regions face exceptionally urgent and complex challenges, demanding innovative solutions and optimized existing systems. Our research reveals a distinct, heterogeneous relationship between the SDI and the ASR: regions with high SDI exhibit a lower ASR trend, whereas regions with low-middle SDI display higher ASR characteristics. This finding underscores the distinct impact of socioeconomic status on the distribution of health indicators. Regions with low SDI often face more complex challenges, including uneven distribution of medical resources, unequal access to healthcare information, barriers to healthcare services, inadequate implementation of medical practice standards, and ineffective public health policies. In contrast, high SDI regions allocate superior healthcare resources and deploy specialized medical teams, who provide personalized, systematic, and efficient diagnosis and treatment for encephalitis patients. These actions facilitate precise disease management and long-term control, significantly reducing the chronic health burden. Existing literature indicates that global healthcare expenditure levels and the prevalence of imaging diagnostic equipment exhibit a marked heterogeneous distribution (21). High-income and upper-middle-income countries hold a distinct advantage in diagnostic imaging equipment. They typically provide 5 to 10 CT scanners per million residents. In contrast, low-middle and low-income countries face significant shortages, with an average of 0 to 5 such devices per million residents (22). As a result, low and middle-income countries face severe limitations in implementing optimal management practices for encephalitis. These challenges arise due to a lack of specialized medical equipment needed for specific diagnostics and assessments. This equipment shortage significantly impedes effective disease prevention, control, and treatment. Encephalitis is a complex, severe clinical condition with diverse evolutionary trajectories. Accurate diagnosis and effective treatment rely heavily on advanced expertise and technical capabilities from neurological specialists. This ensures patients receive timely, precise, and appropriate interventions. In low- to middle-income or resource-constrained regions, the availability of neurological professionals falls short of clinical demand. Statistics indicate a median of 0.43 adult neurologists per 100,000 population globally. By contrast, high-income countries approach five neurologists per 100,000 residents (23). This comparison highlights a substantial gap in medical human resources in low-income and resource-constrained regions, posing significant challenges for diagnosing and treating complex diseases such as encephalitis. Taking the Philippines as an example, a survey indicates that one adult neurologist serves approximately 176,000 Filipino adults. This amounts to 0.57 adult neurologists per 100,000 adult population. Meanwhile, pediatric neurologists are even more scarce. One pediatric neurologist serves 299,581 Filipino children, or 0.33 pediatric neurologists per 100,000 children (24). These statistics reveal significant disparities in the allocation of healthcare resources. Adequate medical staffing is crucial for improving the diagnosis and treatment outcomes of patients with encephalitis. It also facilitates their recovery process. The shortage of neurologists may worsen patients’ inability to access timely and appropriate evaluation and treatment. This not only affects their health status but also increases the risk of severe complications. To improve encephalitis treatment outcomes, especially in low-income countries and regions, regional interdisciplinary collaboration is urgently needed. This includes a focus on training healthcare personnel, capacity development, and strengthening treatment strategies. Such initiatives aim to consolidate resources, optimize service delivery, and ensure effective responses to encephalitis challenges in these regions (25). At the same time, to alleviate the global public health burden caused by encephalitis, the international community should strengthen cooperative frameworks and focus resources on supporting the development of healthcare systems in affected areas. This will help ensure all patients receive timely, high-quality medical care.

Since its initial recognition, autoimmune encephalitis (AE) has been established as a dynamically evolving neurological disorder (26). Over the past decade, research on AE has advanced rapidly. This progress is driving continuous changes in clinical diagnosis and treatment approaches (27). Recent advances in autoantibody detection technologies have revealed that its prevalence now rivals that of infectious encephalitis. This dramatic increase demands corresponding efforts from clinicians to keep pace with emerging knowledge. However, disparities in access to medical information remain particularly pronounced. This is especially true in low and middle-income countries, where conducting high-quality research is often hindered by insufficient capacity. Most patients with AE respond positively to immunotherapy. In some cases, however, prolonged hospitalization and intensive care support may be required to manage their complex conditions. At the same time, the medical burden of patients exhibiting encephalitis-induced behavioral disturbances and resulting long-term disabilities inevitably places significant strain on social welfare systems and family finances. Therefore, quantifying the economic costs associated with the rational allocation of healthcare resources is a critical consideration (28–31). From a long-term perspective, strengthening capacity building in AE research within low and middle-income countries significantly improves patients’ quality of life. It also helps alleviate the economic burdens on society and families. This process requires collaboration among multiple stakeholders and systematic planning. International organizations and philanthropic foundations should prioritize funding and technical guidance to support AE research in these countries. Establishing close partnerships and training local researchers can enhance their professional skills and research standards. This enables them to join the global scientific community and supports international knowledge sharing and collaboration. Secondly, high-income countries should regularly host international academic conferences and symposiums. These events facilitate knowledge exchange among global research communities, promoting the sharing of practical experiences and expertise. Such gatherings offer researchers from low- and middle-income countries the opportunity to meet with leading international experts. They can learn cutting-edge research techniques and gain new insights, which enhances their own research skills and sparks innovation.

Vaccination plays a crucial role in pre-exposure prophylaxis against specific viral encephalitis, particularly for viruses such as tick-borne encephalitis, rabies, and Japanese encephalitis. However, vaccine development and production are time-consuming and complex, requiring advanced technological support and substantial government funding (32). Despite effective vaccines existing, low and middle-income countries face several key challenges in implementing vaccination programs: (1) low levels of education result in limited vaccine knowledge and skepticism; (2) severe economic constraints and insufficient healthcare infrastructure mean fewer accessible vaccination sites, especially in rural areas; (3) the high cost and multiple doses of vaccines place additional strain on healthcare systems. These barriers significantly hinder vaccination uptake in these settings (33). Secondly, for low and middle-income countries, major challenges in vaccine supply include, but are not limited to, the lack of clinical trial infrastructure, deficiencies in the effectiveness of the regulatory system, and the absence of sustainable financing channels (34). These systemic barriers not only limit the widespread adoption of vaccines but also impose enduring constraints on vaccine program development, severely undermining their long-term sustainability. We must urgently implement comprehensive and targeted public health interventions in low and low-middle SDI regions to reduce key risk factors that exacerbate the burden of encephalitis. We should strengthen health education and awareness campaigns on vaccination in these regions to improve public understanding, dispel misconceptions, and increase vaccine acceptance. This will create a more favorable environment for vaccination. Additionally, systematic investment and optimization of basic health infrastructure in low-income countries are vital. Measures such as increasing vaccination sites and offering mobile services help ensure timely access to vaccinations. Furthermore, increased investment in global health, especially vaccine research and development for low-income countries, is crucial for advancing health equity. By promoting vaccines tailored to regional needs, every country can access products that are specifically suited to address its unique health challenges. These actions optimize limited resources and systematically enhance encephalitis prevention, ultimately upgrading public health systems.

Meanwhile, our research reveals that encephalitis cases are particularly concentrated in Asian countries such as Pakistan, India, Bhutan, and Nepal, where public health systems face severe challenges. This is manifested in high prevalence, incidence, and mortality, as well as a significant increase in DALYs. Therefore, these nations should be regarded as key focal areas for future monitoring of the encephalitis disease burden. Persistently high incidence and mortality rates in South and Southeast Asian countries likely result from factors such as high population density, inadequate public health infrastructure, and limited vaccine coverage (10). Japanese Encephalitis (JE), a mosquito-borne viral disease caused by the Japanese Encephalitis Virus (JEV), is primarily endemic in Southeast Asia, India, Indonesia, and parts of Australia (35). Currently, there is no specific treatment for Japanese encephalitis, so existing strategies focus on supportive and symptomatic care. Clinically validated, safe, and effective vaccines exist and have been approved to prevent Japanese encephalitis virus infection. Widespread vaccination has significantly reduced disease incidence and mortality, particularly among high-risk groups, such as children, underscoring the importance of vaccines in public health strategies. However, in recent years, the geographic distribution of Japanese encephalitis in Nepal has expanded, accompanied by complex challenges. Key factors behind its emergence and recurrence include low vaccination coverage, unvaccinated swine populations, limited public health awareness, inadequate mosquito control, vector spread facilitated by climate change, poor sanitation, and the use of irrigated rice farming systems (36). Notably, since China incorporated the Japanese encephalitis vaccine into its immunization program in 2008, a two-dose regimen for children under 15 years of age has led to a decline in disease incidence, from 1.63 per 100,000 in 2004 to 0.16 per 100,000 in 2018 (37). China’s rapid economic growth and major improvements in public health have positively influenced living conditions. Key measures include purifying sewage to reduce mosquito breeding, optimizing pig farming to minimize the transmission of Japanese encephalitis virus, and relocating pigsties away from homes to lower risks (38). These steps have helped curb the spread of Japanese encephalitis. Asian nations like Pakistan, India, Bhutan, and Nepal can learn from China’s success. By boosting vaccination rates and implementing targeted public health strategies, they can reduce disease transmission and mitigate its socioeconomic impact. Expanding access to water, sanitation, and hygiene (WASH) services also remains central to effective disease prevention (39). In South Asia, particularly in Afghanistan, Bangladesh, Nepal, and Pakistan, significant improvements have been observed in enhancing access to water and sanitation services. However, over 134 million people in the region still lack access to safe drinking water, with contamination levels ranging from 68 to 84% (40). Statistical data indicate that approximately 52% of households in rural Pakistan lack sanitation facilities, while this proportion drops significantly to 8% in urban areas (41). This contrast reveals substantial disparities in basic infrastructure coverage between urban and rural areas.

Our analysis reveals significant variations in disease burden across different age groups. This underscores not only the importance of personalized intervention strategies but also the need for precision medicine measures tailored to specific age cohorts. In statistical analyses spanning 1990 to 2021, we found that the older adult consistently ranked highest in ASMR. In contrast, children exhibited markedly higher ASIR and ASDR. These findings illustrate disparities in health and quality of life across different life stages. Furthermore, multiple studies have validated age as a key independent risk factor, revealing its significant impact on fatal outcomes associated with encephalitis. In particular, the higher mortality rate among the older adult is typically attributed to the aging of the immune system and the interaction of multiple comorbidities (42). The incidence of cardiovascular and cerebrovascular diseases rises significantly in the older adult, along with a marked decline in immune function. When pathogens invade the nervous system, the older adult struggle to activate mechanisms that secrete cytokines, chemokines, and specific antibodies. These responses are crucial in regulating inflammation. As a result, the older adult are at a disadvantage in fighting neurological infections. Additionally, cerebrospinal fluid changes in the older adult lack specificity, and imaging tests such as cranial MRI and Electroencephalogram (EEG) have low detection rates. These factors can lead to missed diagnoses and affect disease progression. Encephalitis is also common in children. An Australian multicenter study found acute encephalitis to be more prevalent in young children, with a median age of 1.7 years (43). The higher incidence and mortality rates in children stem from their fast metabolism and immature internal buffering and neuroendocrine systems. Children resist external environmental stimuli less, making them more susceptible to internal imbalances that can exacerbate disease progression and impede recovery. This likely connects to their underdeveloped blood–brain barrier and atypical clinical symptoms. Regular health monitoring and rapid identification of infection signs in vulnerable groups are key to protecting health. Current encephalitis research shows males have a higher incidence of encephalitis than females. Further analysis using Anti-NMDA receptor encephalitis criteria reveals that females comprise 64% of cases (44), underscoring their predominance in this disease. Another French study on HSV encephalitis enrolled 1,425 patients with a male-to-female ratio of 1.07 (736/689), indicating a slight predominance of males (45). These observations suggest that clinical manifestations of encephalitis may exhibit significant heterogeneity across different subtypes, a phenomenon potentially linked to the complex diversity of genetic backgrounds or intrinsic differences in disease pathogenesis.

## Conclusion

This study provides valuable data on encephalitis diseases, which have significant implications for global public health. To mitigate the impact of encephalitis at the global, regional, and national levels, it is essential to develop targeted intervention policies for high-risk areas and populations, with a focus on prevention and treatment. As the world’s population continues to grow, more attention must be given to addressing this urgent issue.

### Limitation

First, as this study is a secondary analysis of GBD research data, the accuracy and reliability of the estimates are limited. Many low- and middle-income countries, notably in Africa and South Asia, lack adequate disease surveillance and consistent healthcare reporting, resulting in missing or incomplete data. Consequently, encephalitis cases may be underreported in disadvantaged regions, causing the disease burden to be underestimated. Second, the GBD 2021 database does not classify encephalitis by etiology, so this study was unable to differentiate between infectious and immune-mediated types or identify post-infectious cases. This omission prevents analysis of subtype-specific burdens and variations. Future studies should gather detailed etiological data to clarify the epidemiology of different causative agents. Third, rapid changes in encephalitis epidemiology, driven by socioeconomic and environmental factors, may not be fully reflected in GBD data despite its regular updates.

## Data Availability

The original contributions presented in the study are included in the article/supplementary material, further inquiries can be directed to the corresponding author.

## Footnotes

1
https://ghdx.healthdata.org/gbd-2021/sources

2
https://gbd2021.healthdata.org/gbd-results/

3
https://ghdx.healthdata.org/record/global-burden-disease-study-2021-GBD-2021-socio-demographic-index-sdi-1950-2021

## References

[1] Glaser CA Bloch KC. Encephalitis: a global problem deserving of a global approach. Clin Infect Dis. (2020) 70:2527–9. doi: 10.1093/cid/ciz690, 31549167

[2] Alam AM Easton A Nicholson TR Irani SR Davies NWS Solomon T . Encephalitis: diagnosis, management and recent advances in the field of encephalitides. Postgrad Med J. (2023) 99:815–25. doi: 10.1136/postgradmedj-2022-141812, 37490360

[3] Granerod J Huang Y Davies NWS Sequeira PC Mwapasa V Rupali P . Global landscape of encephalitis: key priorities to reduce future disease burden. Clin Infect Dis. (2023) 77:1552–60. doi: 10.1093/cid/ciad417, 37436770 PMC10686956

[4] Wesselingh R Griffith S Broadley J Tarlinton D Buzzard K Seneviratne U . Peripheral monocytes and soluble biomarkers in autoimmune encephalitis. J Autoimmun. (2023) 135:103000. doi: 10.1016/j.jaut.2023.103000, 36753921

[5] Fouque F Reeder JC. Impact of past and on-going changes on climate and weather on vector-borne diseases transmission: a look at the evidence. Infect Dis Poverty. (2019) 8:51. doi: 10.1186/s40249-019-0565-1, 31196187 PMC6567422

[6] Khandaker G Jung J Britton PN King C Yin JK Jones CA. Long-term outcomes of infective encephalitis in children: a systematic review and meta-analysis. Dev Med Child Neurol. (2016) 58:1108–15. doi: 10.1111/dmcn.13197, 27422743

[7] International Encephalitis. Encephalitis in adults – a guide. Malton: Encephalitis International (2024).

[8] Rubin R. Profile: Institute for Health Metrics and Evaluation, WA, USA. Lancet. (2017) 389:493. doi: 10.1016/S0140-6736(17)30263-5, 28170325

[9] GBD 2019 Diseases and Injuries Collaborators. Global burden of 369 diseases and injuries in 204 countries and territories, 1990-2019: a systematic analysis for the global burden of disease study 2019. Lancet. (2020) 396:1204–22. doi: 10.1016/S0140-6736(20)30925-9, 33069326 PMC7567026

[10] Wang H Zhao S Wang S Zheng Y Wang S Chen H . Global magnitude of encephalitis burden and its evolving pattern over the past 30 years. J Infect. (2022) 84:777–87. doi: 10.1016/j.jinf.2022.04.026, 35452715

[11] GBD 2021 Diseases and Injuries Collaborators. Global incidence, prevalence, years lived with disability (YLDs), disability-adjusted life-years (DALYs), and healthy life expectancy (HALE) for 371 diseases and injuries in 204 countries and territories and 811 subnational locations, 1990-2021: a systematic analysis for the global burden of disease study 2021. Lancet. (2024) 403:2133–61. doi: 10.1016/S0140-6736(24)00757-8, 38642570 PMC11122111

[12] Chen X Xiang X Xia W Li X Wang S Ye S . Evolving trends and burden of inflammatory bowel disease in Asia, 1990-2019: a comprehensive analysis based on the global burden of disease study. J Epidemiol Glob Health. (2023) 13:725–39. doi: 10.1007/s44197-023-00145-w, 37653213 PMC10686927

[13] Long H Liu Q Yin H Wang K Diao N Zhang Y . Prevalence trends of site-specific osteoarthritis from 1990 to 2019: findings from the global burden of disease study 2019. Arthritis Rheumatol. (2022) 74:1172–83. doi: 10.1002/art.42089, 35233975 PMC9543105

[14] Kim HJ Fay MP Feuer EJ Midthune DN. Permutation tests for joinpoint regression with applications to cancer rates. Stat Med. (2000) 19:335–51. doi: 10.1002/(sici)1097-0258(20000215)19:3<3.0.co;2-z, 10649300

[15] Kumar R. Understanding and managing acute encephalitis. F1000Res. (2020) 9:F1000. doi: 10.12688/f1000research.20634.1, 32047620 PMC6993835

[16] Whitley RJ Lakeman F. Herpes simplex virus infections of the central nervous system: therapeutic and diagnostic considerations. Clin Infect Dis. (1995) 20:414–20. doi: 10.1093/clinids/20.2.414, 7742450

[17] Hjalmarsson A Blomqvist P Sköldenberg B. Herpes simplex encephalitis in Sweden, 1990-2001: incidence, morbidity, and mortality. Clin Infect Dis. (2007) 45:875–80. doi: 10.1086/521262 17806053

[18] Piantadosi A Mukerji SS Ye S Leone MJ Freimark LM Park D . Enhanced virus detection and metagenomic sequencing in patients with meningitis and encephalitis. MBio. (2021) 12:e0114321. doi: 10.1128/mBio.01143-21, 34465023 PMC8406231

[19] Upreti SR Lindsey NP Bohara R Choudhary GR Shakya S Gautam M . Updated estimation of the impact of a Japanese encephalitis immunization program with live, attenuated SA 14-14-2 vaccine in Nepal. PLoS Negl Trop Dis. (2017) 11:e0005866. doi: 10.1371/journal.pntd.0005866, 28934197 PMC5608168

[20] Streng A Grote V Rack-Hoch A Liese JG. Decline of neurologic varicella complications in children during the first seven years after introduction of universal varicella vaccination in Germany, 2005-2011. Pediatr Infect Dis J. (2017) 36:79–86. doi: 10.1097/inf.0000000000001356 27749651

[21] Alberto NRI Alberto IRI Puyat CVM Antonio MAR Ho FDV Dee EC . Disparities in access to cancer diagnostics in ASEAN member countries. Lancet Reg Health West Pac. (2023) 32:100667. doi: 10.1016/j.lanwpc.2022.100667, 36785859 PMC9918780

[22] Brown A Batra S. Rare hematologic malignancies and pre-leukemic entities in children and adolescents young adults. Cancers (Basel). (2024) 16:997. doi: 10.3390/cancers16050997, 38473358 PMC10930531

[23] Organization WH. Atlas country resources for neurological disorders. Geneva: World Health Organization (2017).

[24] Association PN. PNA Fellows (2021). Available online at: https://www.philippineneurologicalassociation.com/pan-fellows (Accessed September 3, 2021).

[25] Ortiz R Vásquez L Giri B Kapambwe S Dille I Mahmoud L . Developing and sustaining high-quality care for children with cancer: the WHO global initiative for childhood cancer. Rev Panam Salud Publica. (2023) 47:e164. doi: 10.26633/rpsp.2023.164, 38116183 PMC10729910

[26] Graus F Titulaer MJ Balu R Benseler S Bien CG Cellucci T . A clinical approach to diagnosis of autoimmune encephalitis. Lancet Neurol. (2016) 15:391–404. doi: 10.1016/S1474-4422(15)00401-9, 26906964 PMC5066574

[27] Dubey D Pittock SJ Kelly CR McKeon A Lopez-Chiriboga AS Lennon VA . Autoimmune encephalitis epidemiology and a comparison to infectious encephalitis. Ann Neurol. (2018) 83:166–77. doi: 10.1002/ana.25131, 29293273 PMC6011827

[28] Titulaer MJ McCracken L Gabilondo I Armangué T Glaser C Iizuka T . Treatment and prognostic factors for long-term outcome in patients with anti-NMDA receptor encephalitis: an observational cohort study. Lancet Neurol. (2013) 12:157–65. doi: 10.1016/s1474-4422(12)70310-1, 23290630 PMC3563251

[29] Newman MP Blum S Wong RC Scott JG Prain K Wilson RJ . Autoimmune encephalitis. Intern Med J. (2016) 46:148–57. doi: 10.1111/imj.12974, 26899887

[30] Mittal MK Rabinstein AA Hocker SE Pittock SJ Wijdicks EF McKeon A. Autoimmune encephalitis in the ICU: analysis of phenotypes, serologic findings, and outcomes. Neurocrit Care. (2016) 24:240–50. doi: 10.1007/s12028-015-0196-8, 26319044

[31] Lee WJ Lee ST Moon J Sunwoo JS Byun JI Lim JA . Tocilizumab in autoimmune encephalitis refractory to rituximab: an institutional cohort study. Neurotherapeutics. (2016) 13:824–32. doi: 10.1007/s13311-016-0442-6, 27215218 PMC5081109

[32] Bedford H Attwell K Danchin M Marshall H Corben P Leask J. Vaccine hesitancy, refusal and access barriers: the need for clarity in terminology. Vaccine. (2018) 36:6556–8. doi: 10.1016/j.vaccine.2017.08.004 28830694

[33] Branda F Ali AY Ceccarelli G Albanese M Binetti E Giovanetti M . Assessing the burden of neglected tropical diseases in low-income communities: challenges and solutions. Viruses. (2024) 17:29. doi: 10.3390/v1701002 39861818 PMC11769400

[34] Thobari JA Arguni E Bunoan-Macazo JA Clark S Dorj N Douangboupha V . Opportunities and challenges of conducting vaccine research in low and middle-income countries in the Asia-Pacific region: perspectives from the Asia-Pacific vaccine research network. Lancet Reg Health Am. (2025) 58:101559. doi: 10.1016/j.lanwpc.2025.101559, 40475887 PMC12140067

[35] Barrows NJ Campos RK Liao KC Prasanth KR Soto-Acosta R Yeh SC . Biochemistry and molecular biology of Flaviviruses. Chem Rev. (2018) 118:4448–82. doi: 10.1021/acs.chemrev.7b00719, 29652486 PMC5937540

[36] Ghimire S Dhakal S. Japanese encephalitis: challenges and intervention opportunities in Nepal. Vet World. (2015) 8:61–5. doi: 10.14202/vetworld.2015.61-65, 27046998 PMC4777813

[37] Gao X Li X Li M Fu S Wang H Lu Z . Vaccine strategies for the control and prevention of Japanese encephalitis in mainland China, 1951-2011. PLoS Negl Trop Dis. (2014) 8:e3015. doi: 10.1371/journal.pntd.0003015, 25121596 PMC4133196

[38] Chen XJ Wang HY Li XL Gao XY Li MH Fu SH . Japanese encephalitis in China in the period of 1950-2018: from discovery to control. Biomed Environ Sci. (2021) 34:175–83. doi: 10.3967/bes2021.024 33766213

[39] UNICEF. Progress on household drinking water, sanitation and hygiene, 2000–2020: five years into the SDGs. Geneva: World Health Organization (2021).

[40] Parry CM Ribeiro I Walia K Rupali P Baker S Basnyat B. Multidrug resistant enteric fever in South Asia: unmet medical needs and opportunities. BMJ. (2019) 364:k5322. doi: 10.1136/bmj.k5322 30670452 PMC6340381

[41] Khan AY Fatima K Ali M. Sanitation ladder and undernutrition among under-five children in Pakistan. Environ Sci Pollut Res Int. (2021) 28:38749–63. doi: 10.1007/s11356-021-13492-7, 33740192

[42] Miller RA. The aging immune system: primer and prospectus. Science. (1996) 273:70–4. doi: 10.1126/science.273.5271.70, 8658199

[43] Britton PN Dale RC Blyth CC Clark JE Crawford N Marshall H . Causes and clinical features of childhood encephalitis: a multicenter, prospective cohort study. Clin Infect Dis. (2020) 70:2517–26. doi: 10.1093/cid/ciz685, 31549170

[44] Alsalek S Schwarzmann KB Budhathoki S Hernandez-Lopez V Smith JB Li BH . Racial and ethnic disparities in the incidence of anti-NMDA receptor encephalitis. Neurol Neuroimmunol Neuroinflamm. (2024) 11:e200255. doi: 10.1212/NXI.0000000000200255, 38728608 PMC11089538

[45] Sauvage A Laurent E Gaborit C Guillon A Grammatico-Guillon L. Herpes simplex encephalitis in France: incidence, 6-month rehospitalizations and mortality. Infection. (2024) 52:1965–72. doi: 10.1007/s15010-024-02272-3, 38678152

